# Editorial: Advanced EEG analysis techniques for neurological disorders

**DOI:** 10.3389/fninf.2025.1637890

**Published:** 2025-06-18

**Authors:** Jisu Elsa Jacob, Sreejith Chandrasekharan

**Affiliations:** ^1^Department of Electronics and Communication Engineering, Sree Chitra Thirunal College of Engineering, Trivandrum, Kerala, India; ^2^Freelance Researcher, Trivandrum, Kerala, India

**Keywords:** electroencephalogram (EEG), large language model (LLM), neurological disorders, visualization of EEG, Brain computer interface (BCI)

## 1 Introduction

Electroencephalogram or EEG analysis has undergone a profound transformation with the addition of advanced artificial intelligence and computational methods to the domain. Contemporary EEG analysis expands beyond traditional methods that relied on visual inspection, employing refined algorithms that are capable of detecting subtle patterns that are not evident in time signals and to the human eye. Such state-of-the-art techniques include artificial intelligence, advanced statistical methods, and sophisticated signal processing approaches to unlock deeper insights into brain signals and dysfunctions. From using large language models (LLMs) for enhanced diagnostic interpretation to real-time Brain-Computer Interfaces (BCI), recent research has pushed the boundaries of EEG signal analysis.

## 2 Discussion of recent research in advanced EEG analysis

The technical summaries that follow are intended to provide an overview of recent and impactful peer-reviewed research that illustrates advanced approaches that address critical challenges in neurological care. Each of the topics represents a valuable leap forward in terms of the diagnosis and monitoring of complex neurological conditions with unparalleled precision and efficiency. The research papers included in this Research Topic address the following categories in this area of research.

### 2.1 Foundation AI models for neuroscience

Recent advances in large language models (LLMs) and the application of such models to EEG-based disease diagnostics, by tuning with a vast background knowledge base from neuroscience, disease diagnostics, and signal processing techniques, have helped in the transformation of such systems. Chandrasekharan et al. provide an overview of the context, starting with a comparison of LLMs against traditional neural networks, such as sequence-to-sequence networks, which require large datasets, comprehensive training strategies, and learning parameter adjustments through hyperparameter tuning, demanding expert-level knowledge in Artificial Intelligence (AI). In contrast to such traditional systems, LLMs achieve expert-level performance through minimal training data, minor tuning through prompt engineering, and much less computational requirement, leading to shorter deployment times for highly effective diagnostic solutions. Such diagnostic methods not only aid in disease classification and analysis, but also generate human expert-like reasoning, justifying the decisions they make, which allows for review and further improvements under expert supervision. Optimization of such systems is achieved through Low-Rank Adaptation of Large Language Models (LoRA), addressing bottlenecks introduced by computational requirements. Furthermore, this survey highlights deployment challenges and ethical considerations, and stimulates research in EEG signal analysis through LLMs and related machine learning pipelines.

### 2.2 Brain-computer interface systems

The domain of the brain computer interface (BCI) generally requires a large amount of subject-specific labeled data for the training phase to achieve effective calibration of the models and ensure reliable classification on each new subject. With the motor imagery encephalographic signal analysis system (MI-EEG), the use of extended least squares regression-based inductive transfer learning helps achieve this knowledge transfer from the source domain to the target domain in the event of data insufficiency, as described in the study by Jiang et al.. By this approach, broader applications can be addressed with the inclusion of several classic base models such as neural networks, a custom fuzzy logic approach namely the Takagi-Sugeno-Kang fuzzy system and kernel methods, which can find patterns in complex data. In the context of the classification of physical actions studied by Gordienko et al., a fully connected deep neural network (FCN) in combination with layers of convolutional neural networks (CNN) classifies finger-palm-hand from the grasp-and-lift dataset. This study uses Natural Noise Augmentation (NDA) in contrast to a synthetic approach by increasing the sampling size and using different offset values for labeling introducing Gaussian noise and thus providing improved performance. The study performed with Detrended Fluctuation Analysis (DFA) investigated fluctuation properties and calculated Hurst components that revealed the ability of shorter EEG fragments to demonstrate higher complexity and enabled the system to run on low-resource-requirement systems.

### 2.3 Clinical data integration and visualization tools

EEG reports predominantly exist in unstructured textual formats, complicating data extraction and analysis for large-scale studies. A hierarchical algorithm transforms these reports using natural language processing (NLP) techniques through two phases: deep learning-based text classification followed by rule-based keyword extraction procedures in the study by Chung et al.. The algorithm categorizes reports into normal and abnormal groups, then systematically identifies key indicators of cerebral dysfunction or seizures, distinguishing between focal (localized) and generalized seizures while identifying epileptiform discharges and their anatomical locations. Analysis of 17,172 EEG reports from 3,423 pediatric patients achieved accuracy exceeding 98.5% for seizure type determination and over 88.5% for epileptiform discharge detection. In another study by Evans et al., stereoelectroencephalography (sEEG), combines preimplantation magnetic resonance imaging, post-implant computed tomography for electrode visualization, and temporally recorded electrophysiological data for surgical planning. SEEG4D creates automated containerized pipelines segmenting tissues and electrode contacts, aligning contacts with electrical activity, and animating based on relative power. This generates four-dimensional virtual reality components that allow simultaneous viewing of anatomy and seizure activity with automated contact segmentation within 1mm accuracy.

### 2.4 Disease specific diagnostic applications

The various EEG signal analyses have had a high impact in disease diagnosis and prediction, specifically in neurological diseases such as epilepsy, Alzheimer's disease, and even in the prediction of seizures and the diagnosis of early diseases such as dementia. In this Research Topic, Hernandez et al. analyzed the effects of Multiple sclerosis treatment using sample entropy that can measure signal regularity and Higuchi's fractal dimension that can quantify signal complexity in EEG signals from 175 subjects including Interferon-β (n=39), dimethyl fumarate (n=53), and healthy controls (n=83). Both treatment groups exhibited more complex EEG signals than controls, with sample entropy (SampEn) demonstrating significant sensitivity to treatment effects while Higuchi's fractal dimension (HFD) showed greater sensitivity to temporal changes. Absence seizure classification utilized power-to-power cross-frequency coupling (PPC) analysis in Medvedev et al., measuring interactions between oscillations across different time scales in brain rhythm organization. Stacked Sparse Autoencoder (SSAE) networks trained on coupling matrices between frequencies 2–120 Hz achieved 93.1% sensitivity, 99.5% specificity, and 96.8% overall accuracy. In another study in absence seizure, EEG phase synchronization analysis using wavelet phase Glaba et al., synchronization index with normalized amplitude features detected generalized spike-and-wave discharges with 99.2% identification rate. However, ictal-interictal overlap limited pure synchronization-based detection. In Hadeethi et al., alcoholism detection employed clustering technique-based bootstrap (CT-BS) to model sample selection, covariance matrix eigenvalue methods (Cov-Eig) for feature extraction, and the fruit fly optimization algorithm with radius-margin-based support vector machines (FOA-F-SVM). This approach achieved 99% accuracy in the classification of multichannel EEG signals.

## 3 Conclusion

The papers featured in this Research Topic demonstrate how advanced artificial intelligence and computational methods are revolutionizing EEG-based neurological diagnosis and brain-computer interface technologies. From leveraging foundation AI models like Large Language Models to developing sophisticated disease-specific diagnostic tools, these studies collectively address critical challenges in translating complex brain signals into actionable clinical insights. The integration of transfer learning approaches for brain-computer interfaces, advanced visualization tools for surgical planning, and precision diagnostic applications for conditions ranging from epilepsy to alcoholism illustrates the breadth and depth of current EEG research capabilities.

